# Granulocyte Colony-Stimulating Factor Effectively Mobilizes TCR γδ and NK Cells Providing an Allograft Potentially Enhanced for the Graft-Versus-Leukemia Effect for Allogeneic Stem Cell Transplantation

**DOI:** 10.3389/fimmu.2021.625165

**Published:** 2021-03-10

**Authors:** Lia Minculescu, Henrik Sengelov, Hanne Vibeke Marquart, Lars Peter Ryder, Anne Fischer-Nielsen, Eva Haastrup

**Affiliations:** ^1^Department of Clinical Immunology, Rigshospitalet, Copenhagen University Hospital, Copenhagen, Denmark; ^2^Department of Hematology, Rigshospitalet, Copenhagen University Hospital, Copenhagen, Denmark

**Keywords:** TCRγδ cells, NK cells, G-CSF, stem cell grafts, allogeneic transplantation

## Abstract

Allogeneic hematopoietic stem cell transplantation (HSCT) is a potential cure for patients with hematological malignancies but substantial risks of recurrence of the malignant disease remain. TCR γδ and NK cells are perceived as potent innate effector cells in HSCT and have been associated with post-transplant protection from relapse in clinical studies. Immunocompetent cells from the donor are crucial for patient outcomes and peripheral blood stem cells (PBSC) are being increasingly applied as graft source. G-CSF is the preferential mobilizing agent in healthy donors for PBSC grafts, yet effects of G-CSF on TCR γδ and NK cells are scarcely uncovered and could influence the graft composition and potency of these cells. Therefore, we analyzed T and NK cell subsets and activation markers in peripheral blood samples of 49 donors before and after G-CSF mobilization and—for a subset of donors—also in the corresponding graft samples using multicolor flowcytometry with staining for CD3, CD4, CD8, TCRαβ, TCRγδ, Vδ1, Vδ2, HLA-DR, CD45RA, CD197, CD45RO, HLA-DR, CD16, CD56, and CD314. We found that TCR γδ cells were mobilized and harvested with an efficiency corresponding that of TCR αβ cells. For TCR γδ as well as for TCR αβ cells, G-CSF preferentially mobilized naïve and terminally differentiated effector (TEMRA) cells over memory cells. In the TCR γδ cell compartment, G-CSF preferentially mobilized cells of the nonVδ2 types and increased the fraction of HLA-DR positive TCR γδ cells. For NK cells, mobilization by G-CSF was increased compared to that of T cells, yet NK cells appeared to be less efficiently harvested than T cells. In the NK cell compartment, G-CSF-stimulation preserved the proportion of CD56dim NK effector cells which have been associated with relapse protection. The expression of the activating receptor NKG2D implied in anti-leukemic responses, was significantly increased in both CD56dim and CD56bright NK cells after G-CSF stimulation. These results indicate differentiated mobilization and altering properties of G-CSF which could improve the effects of donor TCR γδ and NK cells in the processes of graft-versus-leukemia for relapse prevention after HSCT.

## Introduction

Allogeneic hematopoietic stem cell transplantation (HSCT) is a potential curative treatment for hematological malignancies however the risk of relapse and the detrimental complication, graft-versus-host disease (GVHD), remains (1). Immunocompetent cells from the donor and appropriate immune reconstitution is essential for optimizing the graft-versus-tumor effect (GVL) in patients transplanted for malignant diseases (2, 3). T-cell receptor (TCR) γδ cells and natural killer (NK) cells are perceived as innate effector cells in the setting of HSCT with the ability of mediating GVL in a non-MHC (major histocompatibility complex) restricted manner without increasing the risk of GVHD (4–7). In addition, TCR γδ cells and NK cells play an important role in the protection against infections, primarily cytomegalovirus (CMV), after HSCT (8, 9). Recently, we found higher doses of TCR γδ cells and NK cells in the stem cell graft and during immune reconstitution to be associated with reduced relapse rates and improved survival in patients after HSCT (10, 11). PBSC are being increasingly applied as graft source for HSCT and granulocyte colony-stimulating factor (G-CSF) is used for mobilization of peripheral blood stem cells in healthy donors (12, 13). Meanwhile, it is well known that G-CSF not only mobilizes CD34-positive progenitor cells but also mature lymphocytes into peripheral blood, and G-CSF stimulation can alter the distribution and cause preferential mobilization of various lymphocyte subsets (14, 15). The effect on NK cells and particularly TCR γδ cells is scarcely investigated. We aimed to investigate the potential effects of G-CSF stimulation on TCR γδ and NK cells which could impact their role in GVL for relapse protection after HSCT. For this purpose, peripheral blood of HSCT donors was characterized by T and NK cell markers of subset, differentiation and activation before and after G-CSF stimulation. Also, for a subset of donors, the PBSC grafts were analyzed to assess the corresponding contents of TCR γδ and NK cells, and the graft distribution was compared to bone marrow (BM) grafts.

## Material and Methods

### Stem Cell Donors and Grafts

Donors and grafts were part of a study approved by the Danish National Committee on Health Research Ethics (H-15005137), and all participants gave written informed consent prior to inclusion in accordance with the Declaration of Helsinki. The study was conducted at Copenhagen University Hospital, Rigshospitalet from October 2015 to November 2018; patients were treated with allogeneic HSCT at the Stem Cell Transplant Unit, Department of Hematology and donor evaluation and leukapheresis were performed at the Cell Therapy Facility, Blood Bank Unit, Department of Clinical Immunology, Copenhagen University, Rigshospitalet.

In this original study, 111 patients receiving PBSC/BM grafts from related/unrelated donors were included for analyses of the impact of TCR γδ and NK cells in grafts and during immune reconstitution on patient outcomes. For patients who had related donors, these donors were included for analyses of pre- and post G-CSF blood samples in addition to their PBSC graft samples. By the time patient inclusion ended, 17 patients with related PBSC donors had been included and data from the corresponding 17 donors (pre- and post G-CSF samples) and their harvested PBSC grafts were included in this study. After patient inclusion was closed, inclusion of related donors for PBSC grafts were continued in order to study the impacts of G-CSF. A total of 53 donors (including those from the original 17 donor/patient pairs) were enrolled for pre- and post G-CSF samples. Four donors had insufficient laboratory data leaving a total of 49 donors for inclusion of pre- and post G-CSF samples in this study. Median donor age was 52 (20–71) years with 23 females and 26 males.

During the time period open for patient inclusion, a total of 14 patients received BM grafts (two related and 12 unrelated); these originated from donors separately from those included in this study and were not G-CSF stimulated. Samples from these 14 BM grafts where included in this study for comparison between PBCS and BM graft types. Patient data (including complete graft data and immune reconstitution) and outcomes from the original study are presented in two previous publications (10, 11).

### Mobilization and Leukapheresis

Donors received filgrastim (Nivestim Hospira UK Limited. Maidenhead, UK) 10 µg/kg/day for 5 days. PBSC grafts were obtained by leukapheresis on the fifth day using automated mononuclear cell procedure using the Spectra Optia cell separator (Terumo BCT Inc, Lakewood, CO, USA).

### Sample Collection

Pre-G-CSF samples were obtained on 2 ml EDTA tubes from peripheral blood in donors at the health examination approximately three weeks before leukapheresis. Post-G-CSF samples were obtained on the morning of day 5 of mobilization immediately prior to day 1 of leukapheresis. Graft samples of 500 µL were obtained from the graft bag and analyzed freshly.

### Flowcytometry Analyses

Samples from donors and stem cell grafts were analyzed using the same method. Absolute concentrations of total CD3, CD4, and CD8 T cells and CD16/CD56 NK cells were evaluated by flow cytometry using BD™ Trucount tubes containing fluorescent beads as an internal standard according to the manufacturer’s instructions (BD Biosciences, San Jose, California). Residual volume was used for immune phenotyping in a 2-tube, stain-lyse,10-color flow cytometry panel developed for the study. For multi-fluorochrome staining, 100 µL of whole blood was labeled for TCRαβ-FITC, TCRγδ-PE, CD4-PerCP-Cy5.5, CD45RA-PE-Cy7, CD197-APC, CD45RO-APC-H7, HLA-DR-V450, CD3-V500, and CD8-BV605 for tube 1, and TCRVδ2-FITC, TCRγδ-PE, TCRVδ1-PE-Cy7, CD314-APC, CD16-APC-H7, CD56-V450, CD3-V500, and CD337-BV605 for tube 2. Samples were analyzed using BD™ FACSCanto flow cytometer with the BD™ FACSDiva software which was also used for data analyses.

### Acquisition, Staining and Subset Definitions

We acquired 200,000 events (all events) for pre-G-CSF samples and 400,000 events for post-G-CSF samples in order to obtain comparable numbers of lymphocytes as the fraction of granulocytes is higher after G-CSF stimulation. Phenotype subset definition and gating strategies are shown in **Table 1**and **Figure 1 in Supplemental Data**. TCRαβ was used as a negative control for defining TCRγδ subtypes. CD3 was used as a negative control for defining NK cell subsets. For NKG2D, the MFI values of the entire population of NK and TCR γδ subsets were used instead of separation into positive/negative populations as the expression is a continuum rather than a dichotomous distribution. For HLA-DR we determined the positive threshold based on CD4 T cells where the dominating population is negative; based on this, we found the identification for positive/negative TCR γδ cells evident in the histogram, as shown in the example in **Figure 2 in the Supplemental Data**.

### Cell Concentrations and Immune Phenotyping

Cell subset fractions and concentrations were calculated from the absolute concentrations of total CD3, CD4, and CD8 T cells and CD16/56 NK cells obtained from the TruCount analyses. For immune characterization, we analyzed fractions of differentiation subsets in terms of naïve (CD45RA+CD197+), central memory (CD45RA-CD197+), effector memory (CD45RA-CD197-) and terminally differentiated effector memory (TEMRA, CD45RA+CD197-) cells of CD4, CD8, and TCR γδ T cells. Based on CD16 and CD56 expression, NK cells at different stages of development were defined (16): immature CD16lowCD56bright (termed CD56bright) cells and mature effector CD16posCD56dim (termed CD56dim) cells. The fractions of the CD56bright and CD56dim subsets within total NK cells together with the fraction of TCR γδ cells within total CD3 T cells and fractions of the Vδ1, Vδ2 and nonVδ1-nonVδ2 within total TCR γδ cells, were also analyzed. The expression of human leucocyte antigen HLA-DR as a marker of activation was analyzed on CD4, CD8, and TCR γδ cells T cells. Expression of the activating receptor NKG2D (CD314) on TCR γδ and NK cells was reported as the mean fluorescence intensity (MFI).

### Statistical Analyses

Lymphocyte subset concentrations and percentages in donor peripheral blood and graft samples were non-normally distributed. Differences in concentrations and percentages of cell subsets in donor samples before and after G-CSF mobilization were analyzed using the Wilcoxon signed-rank test for paired observations. Differences in the expression of NKG2D (MFI-values) were analyzed with the paired samples T-test. For analyzes of correlation between continuous variables in donor peripheral blood before/after G-CSF and corresponding graft samples the Kendall’s tau-b test was used. The Mann-Whitney test was used for analyzing the impact of CMV on the lymphocyte concentration and percentages in donor samples both before and after G-CSF mobilization. This was also used for comparisons of cell distributions in PBSC grafts versus BM grafts.

## Results

### Peripheral Blood Concentrations of T and NK Cells Before and After G-CSF Stimulation

**Table 1** shows concentrations of T and NK cell subsets in donors before and after G-CSF stimulation. There was a significant increase in all subset concentrations ranging from 1.5 to 4 fold. In the differentiation subsets of TCR γδ, CD4 and CD8 T cells, the highest increase was in the naïve and TEMRA subsets while the increase in memory subsets was lower for each subset. The increase in TCR γδ cells was similar to that of TRC αβ cells (CD4 and CD8), and the highest increase in TCR γδ subsets was of the Vδ1 and nonVδ1-nonVδ2 type. The increase in NK cells was 30% higher compared to overall CD3 T cells with the highest increase in the CD56dim subset.

**Table 1:** Lymphocyte subset concentrations before and after G-CSF stimulation in peripheral blood of healthy donors, n=49.

Cell subset	Before G-CSFConcentration, 10^6^/L median (IQR)	After G-CSFConcentration, 10^6^/L median (IQR)	P-value	Fold increase(median)
CD3	1500 (1150-1700)	3000 (2400-3875)	<0.001	2.0
CD4	950 (725-1100)	1950 (1600-2500)	<0.001	2.1
CD8	420 (340-590)	975 (690-1375)	<0.001	2.3
TCR γδ	36 (24-81)	77 (50-143)	<0.001	2.1
Vδ1	9.0 (3.9-22)	23 (9.9-41)	<0.001	2.6
Vδ2	20 (9.4-45)	38 (16-74)	<0.001	1.9
NonVδ1-nonVδ2	1.9 (1.3-4.4)	5.4 (1.9-12)	<0.001	2.8
Naïve TCR γδ	2.0 (0.74-3.2)	4.6 (1.6-7.0)	<0.001	2.3
Central memory TCR γδ	3.4 (2.1-6.1)	5.4 (1.8-9.0)	<0.001	1.6
Effector memory TCR γδ	14 (6.0-25)	22 (9.8-47)	<0.001	1.6
TEMRA γδ	17 (8.1-35)	38 (18-72)	<0.001	2.2
Naive CD4	326 (235-487)	881 (517-1244)	<0.001	2.7
Central memory CD4	389 (299-458)	687 (506-844)	<0.001	1.8
Effector memory CD4	136 (98-192)	341 (197-484)	<0.001	2.5
TEMRA CD4	4.3 (2.0-19)	17 (8.4-73)	<0.001	4.0
Naive CD8	103 (62-153)	269 (158 (400)	<0.001	2.6
Central memory CD8	50 (44-78)	76 (47-103)	<0.001	1.5
Effector memory CD8	129 (91-209)	262 (178-386)	<0.001	2.0
TEMRA CD8	115 (43-211)	329 (130-537)	<0.001	2.9
NK (CD16/56)	200 (160-300)	520 (383-688)	<0.001	2.6
CD56dim	187 (137-286)	461 (350-659)	<0.001	2.5
CD56bright	16 (11-22)	33 (26-49)	<0.001	2.1

P–values from Wilcoxon signed rank test for paired observations. Median fold increase.

### Distribution of T and NK Cell Subsets Before and After G-CSF Stimulation

**Figure 1:**
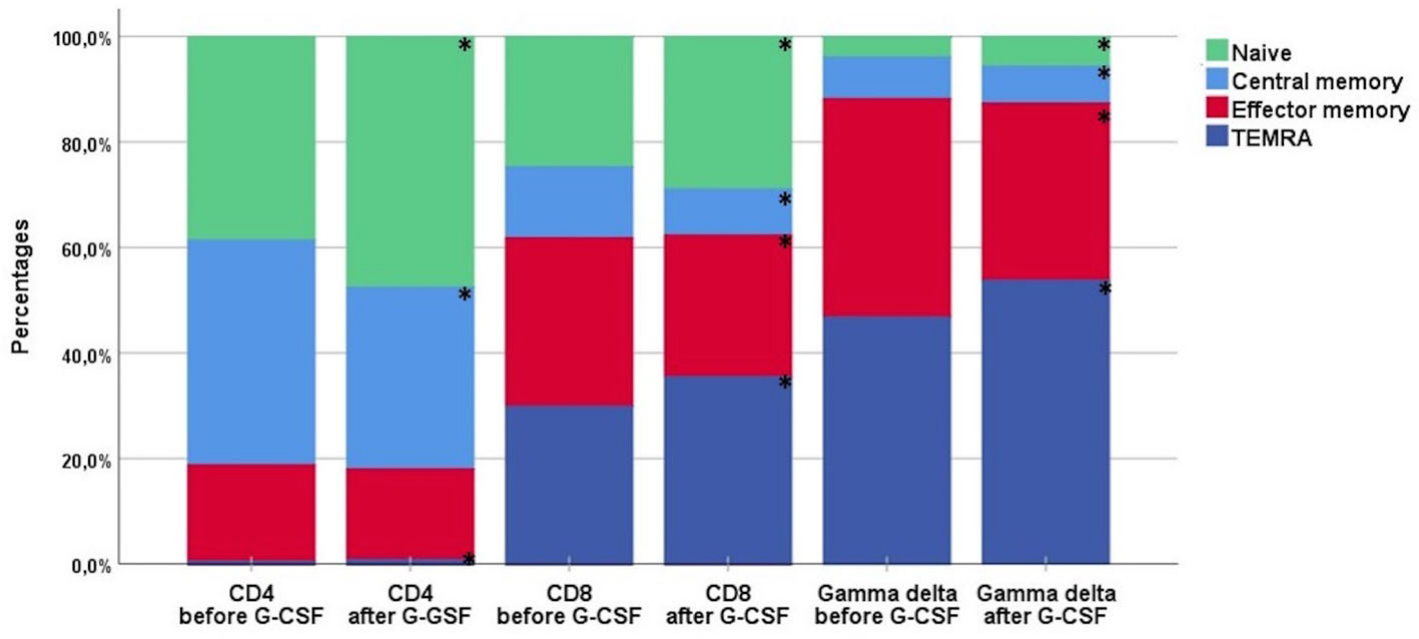
The distribution of differentiation subsets of CD4, CD8, and TCR γδ cells before and after G-CSF stimulation in peripheral blood of healthy donors, n=49. Each compartment is constructed by the use of median percentages. TEMRA; terminally differentiated effector memory. *Statistically significant (p < 0.05) changes in distribution by per cent between before- and after-G-CSF stimulation for each subset.

**Figure 2:**
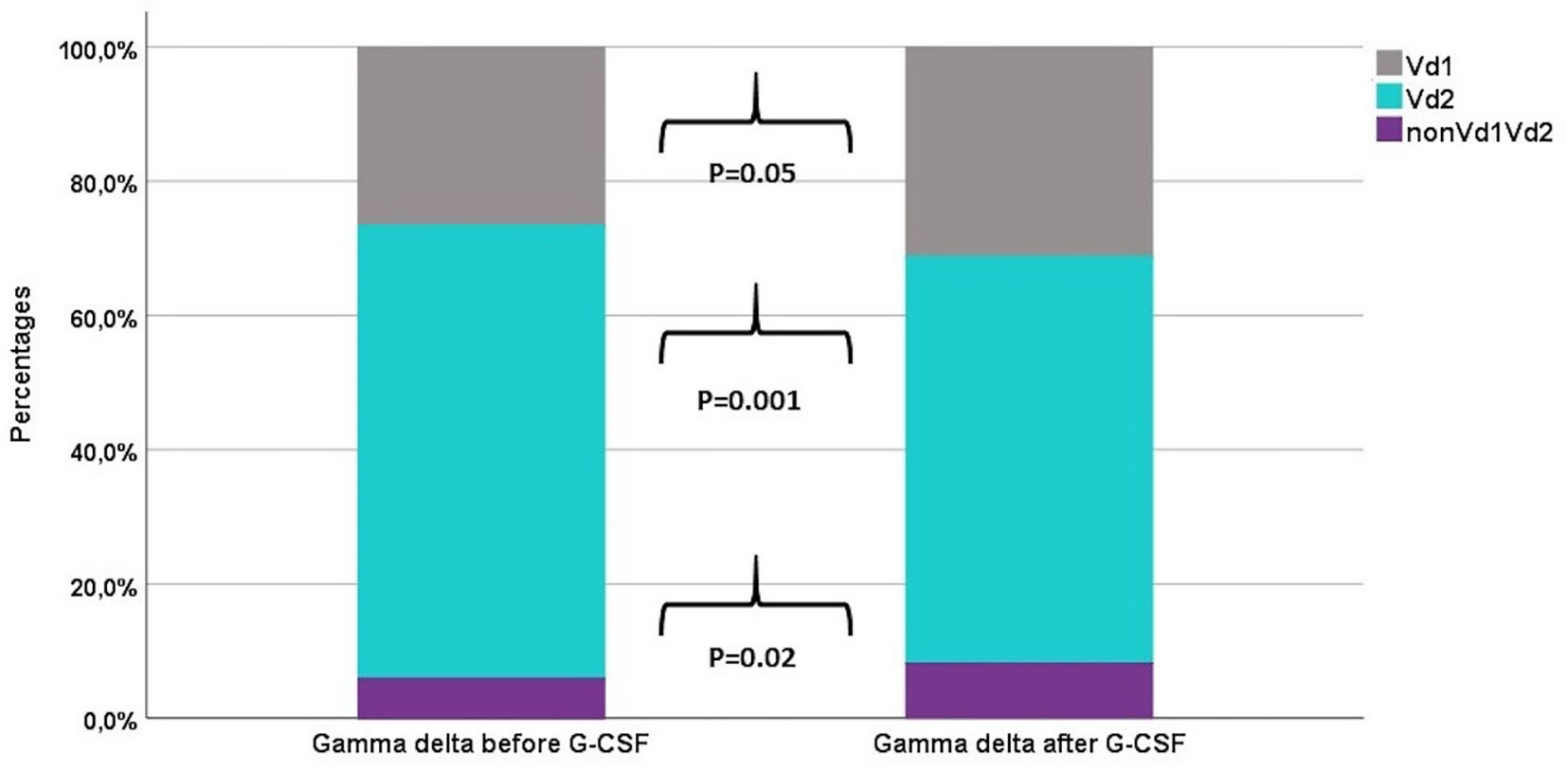
The distribution of TCR γδ cell subsets before and after G-CSF stimulation in peripheral blood of healthy donors, n=49. Each compartment is constructed by the use of median percentages.

**Table 2** shows the percentages of T and NK cell subsets before and after G-CSF stimulation. In the T cell compartment, the fraction of TCR γδ decreased from median 2.6 to 2.4% which was statistically significant in the non-parametric Wilcoxon signed rank test. The ratio between CD4 and CD8 T cells was unaffected. Within the differentiation subsets of TCR γδ, CD4, and CD8 T cells, there was a significant increase in the fraction of both naïve and TEMRA subsets while there was a decrease in the fraction of central memory and effector memory subsets, **Table 2** and **Figure 1**. This corresponded with the higher fold increase of absolute concentrations of naïve and TEMRA cells compared to memory cells, **Table 1**. The fraction of HLA-DR positive cells increased for TCR γδ and CD8 T cells while it decreased for CD4 T cells. In the TCR γδ compartment, the fraction of the Vδ1 and nonVδ1-nonVδ2 subsets increased while the Vδ2 fraction decreased, **Figure 2**, corresponding with the fold increase of absolute concentrations of TCR γδ subsets, **Table 1**. In the NK cell compartment, the subset distribution of the CD56bright and CD56dim subsets remained unaffected as did the total NK cell fraction out of lymphocytes. **Table 2 in Supplemental Data** shows an example of flowcytometry plots of TCR γδ and NK cell subset distribution in donor #46 before- and after G-CSF stimulation.

**Table 2:** Relative concentrations of T and NK cell subsets before and after G-CSF stimulation in peripheral blood of healthy donors, n=49.

Relative concentrations	Before G-CSF	After G-CSF	P-value
CD4/CD3	65 (55-72)	67 (58-73)	0.34
CD8/CD3	31 (26-42)	31 (26-40)	0.41
CD4/CD8, ratio	2.0 (1.4-2.9)	2.1 (1.5-2.9)	0.64
Naïve CD4/CD4	38 (29-49)	47 (31-57)	<0.001
Central memory CD4/CD4	42 (36-53)	34 (29-41)	<0.001
Effector memory CD4/CD4	18 (10-20)	17 (11-25)	0.13
TEMRA CD4/CD4	0.5 (0.2-1.8)	0.8 (0.4-2.5)	0.02
HLA-DR CD4	9.0 (7.6-13)	8.0 (6.0-12)	0.01
Naïve CD8/CD8	24 (12-37)	27 (17-45)	<0.001
Central memory CD8/CD8	13 (9.8-18)	8.2 (5-11)	<0.001
Effector memory CD8/CD8	31 (21-41)	25 (18-35)	<0.001
TEMRA CD8/CD8	29 (12-41)	33 (16-48)	<0.001
HLA-DR CD8	21 (14-28)	23 (18-31)	0.05
Naïve TCR γδ/TCR γδ	3.6 (1.9-8.8)	5.5 (2.8-9.7)	0.01
Central memory TCR γδ/TCR γδ	7.2 (3.9-12)	6.5 (3-11)	0.03
Effector memory TCR γδ/TCR γδ	38 (24-62)	32 (21-45)	0.001
TEMRA TCR γδ/TCR γδ	43 (25-66)	51 (35-71)	0.002
HLA-DR TCR γδ	34 (17-44)	36 (25-49)	0.05
TCR γδ/ CD3 T	2.6 (2.0-5.0)	2.4 (1.5-4.6)	<0.001
Vδ1/ TCR γδ	26 (12-44)	29 (17-49)	0.05
Vδ2/ TCR γδ	66 (50-83)	56 (38-76)	0.001
NonVδ1-nonVδ2/ TCR γδ	5.8 (2.5-18)	7.6 (2.9-16)	0.02
NK(16/56)/lymphocytes	12 (9.1-16)	12 (8.2-15)	0.59
CD56dim/NK	93 (89-95)	94 (91-95)	0.26
CD56bright/NK	6.9 (5.0-11)	6.2 (4.7-9.2)	0.26

Values are given as percent [median (IQR)]. P-values from Wilcoxon signed rank test for paired observations.

### NKG2D Expression Before and After G-CSF Stimulation

**Figure 3:**
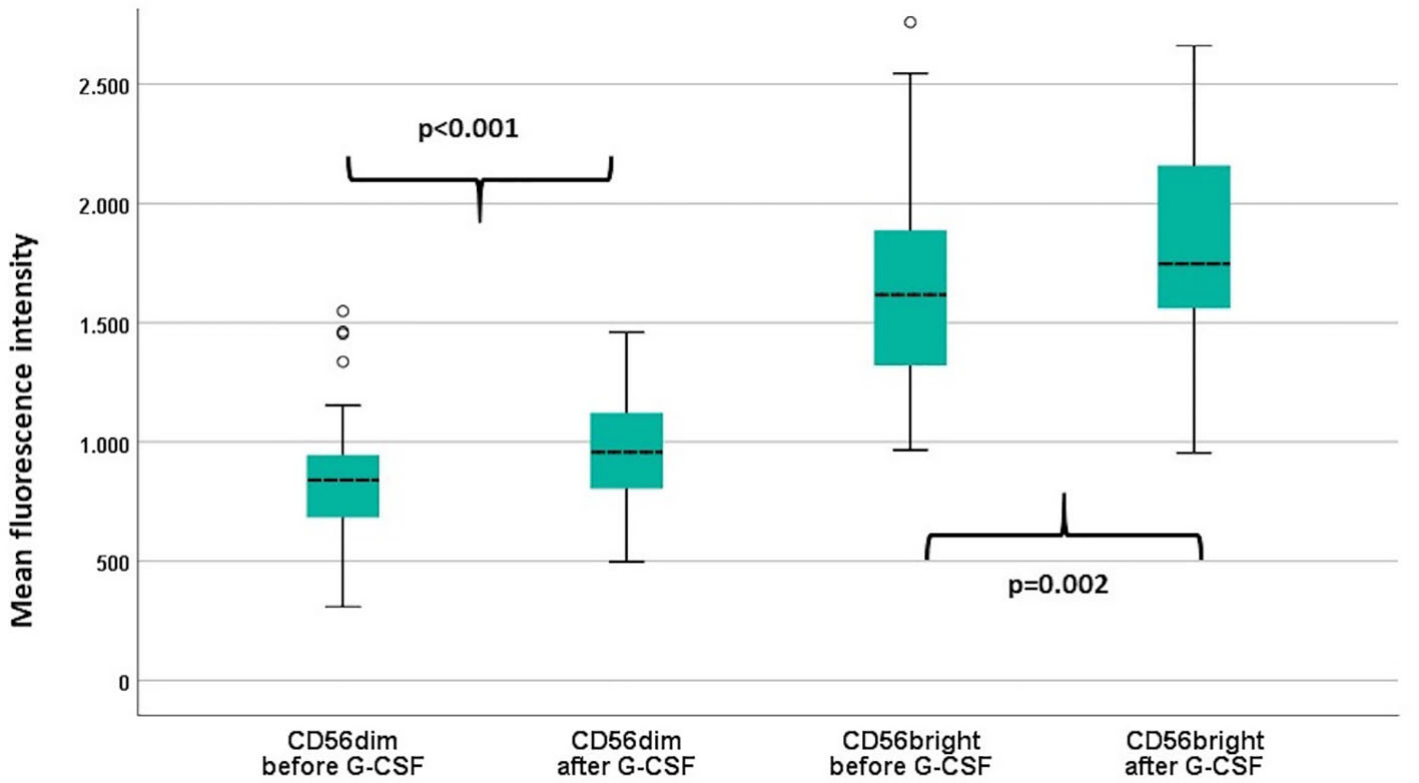
Mean fluorescence intensity (MFI) of NKG2D in the CD56dim and the CD56bright NK cell subsets before and after G-CSF stimulation in peripheral blood of healthy donors, n=49. P-values from paired samples T-test. Boxes represent interquartile range. Whiskers represent minimum and maximum values. Circles represent outliers (1.5 times larger/smaller than the third/first quartile).

We analyzed the expression of the activating receptor NKG2D on TCR γδ and NK cells before and after G-CSF stimulation. The expression was not significantly altered in TCR γδ cells [total TCR γδ cells median MFI 1483 versus 1490 (p=0.16), Vδ1 cells median MFI 1428 versus 1437 (p=0.22), and Vδ2 cells median MFI 1484 versus 1497 (p=0.37)], but was significantly increased after G-CSF in both the CD56bright and CD56dim NK cell subsets, **Figure 3**.

### The Effect of CMV Status on Donor TCR γδ and NK Cells

Out of the 49 donors, 33 (67%) were CMV IgG positive. In peripheral blood samples before G-CSF stimulation, by comparison of groups, CMV positive donors had significantly higher concentrations of Vδ1 (mean 21 vs. 6.6x10^6^/L, p<0.001) and nonVδ1nonVδ2 (mean 6.2 vs.1.7x10^6^/L, p=0.03) cells compared to CMV negative donors. Correspondingly, CMV positive donors had significantly higher fractions of Vδ1 (mean 37 vs. 18%, p=0.006) and lower fractions of Vδ2 cells (mean 52 vs.76%, p=0.01) of out of total TCR γδ cells compared to CMV negative donors. The fraction of effector memory TCR γδ cells was lower (mean 35 vs. 49%, p=0.05) and the fraction of TEMRA TCR γδ cells was higher (mean 53 vs. 32%, p=0.01) in CMV positive compared to negative donors. The overall concentration of TCR γδ cells and fraction of TCR γδ cells out of the total CD3 T cell compartment was not significantly dependent on CMV-status (data not shown). Within the NK cell compartment, there were no differences in the distribution of the CD56dim and CD56bright subsets dependent on CMV status (data not shown), however the MFI expression of NKG2D on both subtypes were higher in CMV positive compared to negative donors (p=0.002 and p=0.04, respectively). Similar differences of subtype distribution according to CMV status was observed in donor samples after G-CSF stimulation (data not shown).

### Correlation Between Graft Doses and Donor Peripheral Blood Concentrations

In 17 donors we analyzed harvested graft contents in addition to peripheral blood concentrations before and after G-CSF stimulation. **Table 3** shows correlations and fold increases of T and NK cell concentrations between pre-G-CSF/post-GSCF and stem cell grafts. All correlations were significant apart from the CD4 T cell and CD56dim NK cell subset (which displayed the same tendency) in donors before G-CSF stimulation and stem cell grafts. A graphical example of correlations for TCR γδ cells are shown in **Supplemental Data, Figure 3**. The increase in concentration before G-CSF stimulation and in harvested stem cell grafts was approximately 20–40 fold and generally higher in T cells compared to NK cells. The highest increase was in the naïve CD8 T cell concentration (56 fold) and the lowest increase in the CD56dim NK cell subset concentration (16 fold). The increase in concentrations from G-CSF-stimulated peripheral blood and to harvested grafts was approximately 10-15-fold in the majority of cell subsets and again slightly lower in NK cells compared to T cells.

**Table 3:** Associations between T and NK cell concentrations in peripheral blood of healthy donors before and after G-CSF stimulation and corresponding concentrations in stem cell grafts, n=17.

Cell subset	Stem cell graft Concentration, 10^9^/L median (IQR)	Correlation to pre-G-CSF concentrations, Rho/p-value	Fold increase to pre-G-CSF concentrations	Correlation to post-G-CSF concentrations, Rho/p-value	Fold increase to post-G-CSF concentrations
CD3	42 (30-59)	0.41/0.03	28	0.43/0.02	14
CD4	26 (20-26)	0.26/0.15	29	0.42/0.02	14
CD8	15 (9.4-22)	0.53/0.003	35	0.50/0.008	15
TCR γδ	0.98 (0.43-1.1)	0.71/<0.001	38	0.62/0.001	16
Vδ1	0.23 (0.11-0.39)	0.65/<0.001	29	0.60/0.001	15
Vδ2	0.34 (0.18-0.62)	0.57/0.001	18	0.73/<0.001	10
NonVδ1-nonVδ2	0.08 (0.04-0.11)	0.69/<0.001	44	0.82/<0.001	16
Naïve TCR γδ	0.04 (0.02-0.07)	0.78/<0.001	36	0.73/<0.001	15
Central memory TCR γδ	0.09 (0.02-0.14)	0.79/<0.001	30	0.72/<0.001	18
Effector memory TCR γδ	0.25 (0.15-0.41)	0.59/0.001	18	0.58/0.002	11
TEMRA γδ	0.37 (0.22-0.58)	0.71/<0.001	26	0.65/<0.001	13
Naive CD4	12 (8.9-19)	0.41/0.02	39	0.67/<0.001	14
Central memory CD4	9.8 (8.2-15)	0.49/0.007	30	0.43/0.02	15
Effector memory CD4	3.2 (1.9-4.8)	0.41/0.02	26	0.37/0.05	14
TEMRA CD4	0.13 (0.09-0.79)	0.62/0.001	37	0.67<0.001	8
Naive CD8	4.6 (2.9-6.0)	0.63/<0.001	56	0.55/0.003	17
Central memory CD8	1.1 (0.9-2.0)	0.41/0.02	23	0.45/0.02	17
Effector memory CD8	3.5 (2.4-6.1)	0.53/0.003	25	0.67/<0.001	15
TEMRA CD8	4.3 (1.1-6.5)	0.69/<0.001	37	0.40/0.03	12
NK cells (CD16/56)	4.6 (3.1-7.7)	0.36/0.05	17	0.37/0.05	10
CD56dim	4.3 (2.9-7.3)	0.33/0.07	16	0.38/0.04	10
CD56bright	0.33 (0.21-0.46)	0.46/0.01	22	0.53/0.004	11

Rho correlation coefficient and p-values from Kendall’s tau-b test for correlation to peripheral blood concentration before and after G-CSF. Median fold increase.

### Comparisons to Bone Marrow Grafts

**Figure 4:**
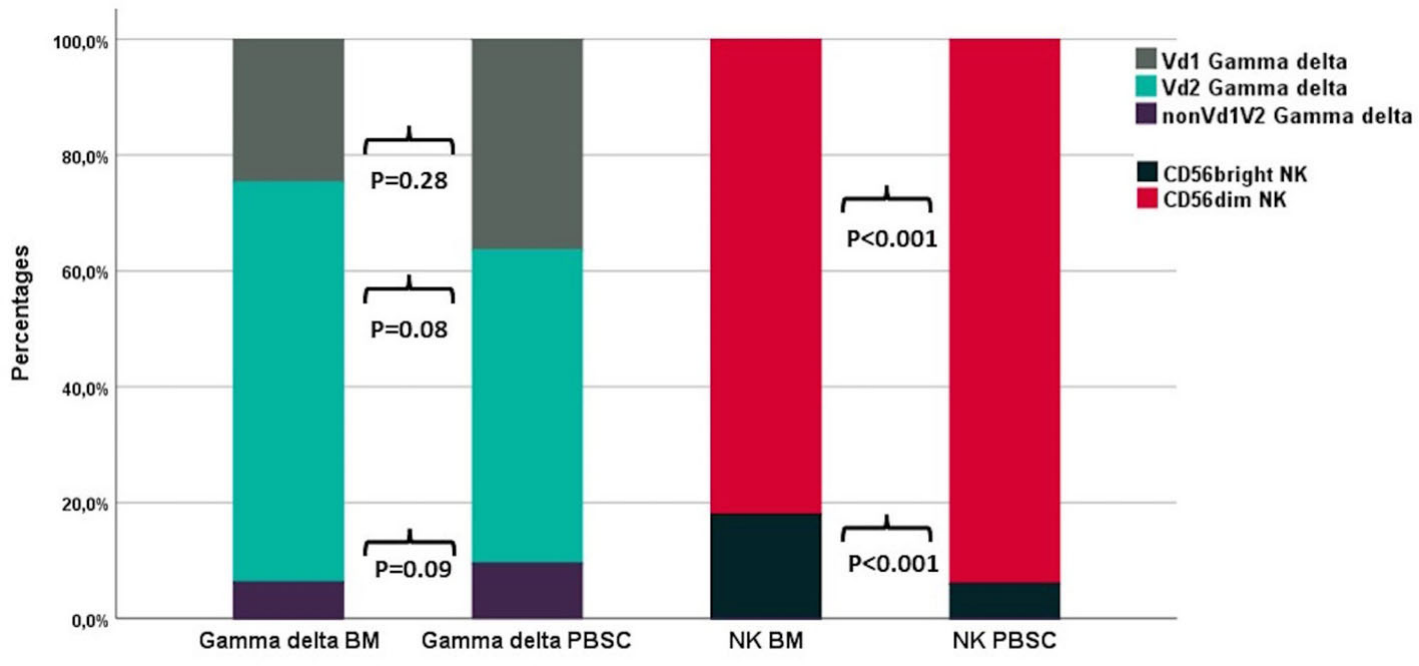
The distribution of TCR γδ and NK cell subsets in BM (n=14) and PBSC (n=17) grafts. P-values from the Mann-Whitney test. Each compartment is constructed by the use of median percentages.

T and NK cell concentrations and distribution in the 17 PBSC grafts were compared with 14 BM grafts. CD34 cell concentrations were median 121 (IQR 60-169) x10^7^/L in PBSC grafts and median 12 (IQR 8.7–17) x10^7^/L in BM grafts. T and NK cell concentrations were median 4,200 (IQR 2,990–5,920) x10^7^/L and median 460 (IQR 310–770) x10^7^/L in PBSC grafts and median 112 (IQR 66–150) x10^7^/L and median 15 (IQR 8.4–21) x10^7^/L in BM grafts. **Table 4** compares T and NK cell subset distributions between graft types. In the T cell compartment, PBSC grafts contained higher fractions of CD4 T cells and lower fractions of both CD8 and TCR γδ T cells compared to BM. Within TCR γδ cells, there was a trend towards lower fractions of Vδ2 cells and higher fractions of nonVδ1nonVδ2 cells in PBSC grafts compared to BM (**Figure 4**). The percentage of HLA-DR positive TCR γδ cells was significantly higher in PBSC compared to BM. In the NK cell compartment, PBSC contained significantly higher fractions of CD56dim cells and lower fractions of CD56bright cells compared to BM (**Figure 4**).

**Table 4:** NK and T cell subset distribution (percent) in stem cell grafts from peripheral blood stem cells (PBSC), n=17, and bone marrow, n=14.

Cell subsets	PBSC median (IQR)	Bone marrow median (IQR)	P-value
CD4/CD3	66 (62-73)	52 (49-57)	<0.001
CD8/CD3	34 (28-39	45 (37-47)	0.001
γδ TCR/CD3	1.6 (1.3-2.5)	4.9 (3.8-6.3)	<0.001
Naïve TCR γδ/TCR γδ	5.4 (2.6-10)	8.7 (3.5-11)	0.32
Central memory TCR γδ/TCR γδ	8.8 (5.2-15)	9.7 (5.2-18)	0.80
Effector memory TCR γδ/TCR γδ	36 (23-49)	39 (29-49)	0.65
TEMRA TCR γδ/TCR γδ	47 (33-56)	37 (24-56)	0.36
HLA-DR TCR γδ/TCR γδ	37 (29-49)	23 (17-26)	0.001
			
Naive CD4/CD4	50 (37-27)	54 (43-60)	0.52
Central memory CD4/CD4	35 (32-47)	29 (26-40)	0.01
Effector memory CD4/CD4	12 (5.9-15)	13 (9.8-20)	0.30
TEMRA CD4/CD4	0.7 (0.3-2.0)	0.6 (0.38-0.73)	0.74
HLA-DR CD4/CD4	8.9 (7.7-12)	7.2 (6.6-9.3.0)	0.02
Naive CD8/CD8	27 (18-44)	49 (32-56)	0.02
Central memory CD8/CD8	12 (6.1-14)	4.6 (3.5-9.5)	0.003
Effector memory CD8/CD8	28 (19-34)	27 (23-37)	0.80
TEMRA CD8/CD8	31 (13-44)	18 (9.2-31)	0.15
HLA-DR CD8/CD8	21 (21-31)	17 (11-30)	0.13
Vδ1/TCR γδ	35 (21-51)	25 (9.0-46)	0.28
Vδ2/TCR γδ	52 (33-69)	70 (43-84)	0.08
nonVδ1nonVδ2/TCR γδ	9.1 (5.8-18)	6.4 (3.4-9.9)	0.09
NK (CD15/56)/lymphocytes	7.9 (6.5-11)	7.4 (5.7-13)	0.83
CD56dim/NK	94 (91-96)	82 (73-91)	<0.001
CD56bright/NK	6.0 (3.8-8.8)	18 (9-26)	<0.001

P-values from Mann-Whitney U test.

The MFI expression of NKG2D was significantly higher in the CD56dim NK cell subset (median 839 vs. 538, p<0.001), the Vδ2 subset (median 1,470 vs. 917, p=0.001) and total TCR γδ cells (median 1,527 vs. 977, p=0.001) in PBSC grafts compared to BM.

## Discussion

In a time of increasing possibilities in individualized treatment options, the effect of mobilizing agents on immunocompetent cells for graft harvest could be a factor in considerations regarding donor selection and transplantation modality. This study reports the effect of G-CSF on concentrations and subset distribution of TCR γδ and NK cells in healthy donors for PBSC-HSCT and correlations of pre-G-CSF values with graft contents.

In our cohort of 49 donors, the concentration of overall TCR γδ cells increased equivalent to TCR αβ T cells after G-CSF mobilization, while the TCR γδ cell subset out of total T cells decreased. Previous studies report conflicting results regarding the effect of G-CSF on the TCR γδ cell fraction (10, 17, 18). Within the total TCR γδ cells, we found a significant increase in the Vδ1 and nonVδ1nonVδ2 subsets and a decrease in the Vδ2 subset similar to changes described by Xuan et al. (19). In humans, the majority of TCR γδ cells in circulation are of the Vδ2 subtype while non-Vδ2 cells, especially Vδ1 cells, are predominant in epithelial tissues (20). Most is known about the anti-tumor properties of Vδ2 cells, however non-Vδ2 cells are gaining increasing attention as effectors of anti-cancer therapy (21) including specific cytotoxicity against hematological malignancies (22, 23) as well as solid tumors (24). Furthermore, non-Vδ2 cells have been shown to have anti-CMV effects and potential cross-reactivity against leukemia cells (25). A specific mobilizing effect of G-CSF of these cell subsets might therefore improve the effect of the TCR γδ compartment towards malignant cells as well as CMV infection after HSCT. Moreover, our findings indicate that G-CSF stimulates TCR γδ activation by increasing the percentages of HLA-DR positive cells. As a part of their adaptive features, TCR γδ cells are able to up-regulate HLA-DR expression upon activation and thereby present antigens to alloreactive TCR αβ cells (26, 27). In HSCT, this mechanism could contribute to GVL (28) why a potentially stimulating effect of G-CSF on HLA-DR expression of TCR γδ could contribute to their anti-leukemic effect in an indirect manner as well.

When applying markers of differentiation similar to those conventionally applied on TCR αβ cells, we found increased subsets of naïve and TEMRA cells and decreased subsets of memory cells within the TCR γδ cell compartment after G-CSF stimulation; similar changes were observed in CD8 T cells. Increases in naïve CD4 and CD8 T cell subsets after G-CSF have previously been described (14), so results from our study indicate a mobilization pattern by G-CSF of naïve TCR γδ cells similar to TCRαβ cells. The role of naïve TCR γδ in GVL or perhaps GVHD is to this point however unknown.

In this study, the overall NK cell concentration increased more than T cell concentrations after G-CSF mobilization, which however was not reflected in changes of the NK cell fraction out of the total lymphocyte count. These findings were in opposition to Melve et al. (14), who found lower NK cell fractions after G-CSF stimulation despite the fact that no changes in the absolute NK cell concentrations was observed in that study. Within NK cell subtypes, smaller studies found an increase in the immature, cytokine-producing CD56bright and a corresponding decrease of the mature, effector CD56dim subset after G-CSF (14, 29). On the contrary, our results indicate preservation of the CD56dim subtype, as the subset distribution was not altered by G-CSF. High doses of CD56dim NK cells in PBSC graft (30) as well as during early immune reconstitution (11) have been associated with reduced relapse rates in clinical studies, indicating a favorable effect of G-CSF in preserving this subset.

Reports investigating the effect of G-CSF on the expression of the NKG2D are scarce; one study reported significant decreased expression of NKG2D on NK cells upon G-CSF stimulation *in vitro (*31). In our study, NKG2D expression was increased in both CD56dim and CD56bright NK cells after G-CSF. In HSCT patients, leukemic cells are able to engage and activate the NKG2D receptor and trigger NK-cell mediated GVL effect through perforin-mediated cytotoxicity, interleukin-priming and activation of costimulatory receptors (32–34). A G-CSF-induced increase in the expression of NKG2D might therefor alter and enhance the anti-leukemic effect of NK-cells in the PBSC transplant setting.

Donors positive for CMV-IgG had higher fractions of nonVδ2 subsets and the TEMRA phenotype of TCR γδ cells compared to CMV-negative donors as previously described in healthy individuals as well as HSCT patients (8, 10). In the NK cell compartment, we observed equal distribution of CD56brigth and CD56dim subsets according to CMV status, however the NKG2D expression was significantly increased in CMV-positive compared to CMV-negative donors for both subsets. Increased NKG2D expression could be a sign of post-infection, long-term activated, memory-like NK cells, as NKG2D is involved in CMV recognition an responses (35, 36). The distribution of T and NK cell subsets and phenotypes in CMV positive compared to negative donors were similar before- and after G-CSF stimulation, indicating mobilizing patterns independent of CMV-status.

In harvested stem cell grafts, the fold increase to pre-GSCF concentrations of TCR γδ cells was higher than that of total CD3 T cells. This suggests an overall mobilization and harvest process equal or possibly superior to that of TCR αβ cells as the fraction of TCR γδ cells out of total T cells in fact significantly decreased after G-CSF stimulation. Interestingly, the Vδ2 subtype had a markedly lower fold increase than the Vδ1 and nonVδ1-nonVδ2 subtypes. Melve et al. (14) also reported a higher fold increase of TCR γδ cells compared to TCR αβ cells after apheresis, but TCR γδ subtyping was not included in that study. Regarding NK cells, the fold increase in grafts compared to pre-G-CSF concentrations was overall lower than that of T cells, presumably as a result of the harvest as the G-CSF mobilization of NK cells was actually higher than that of T cells, Consequently, the harvest of NK cells seemed to be slightly less efficient compared to T cells. Interestingly, this finding is different from one other report (14), and may be due to differences in the apheresis device or settings, which could possibly be optimized to increase the relative collection of NK cells to T cells.

When comparing graft types, the fraction of TCR γδ cells was significantly lower in PBSC compared with BM which was also reported in the 2014 BMTCTN 0201 study including 161 BM grafts and 147 PBSC grafts (37). A higher fraction of potentially anti-leukemic TCR γδ cells in BM grafts could encourage the usage of BM as preferred graft source over PBSC in patients with high-risk or refractory leukemias in cases where additional patient/donor factors allows for it; the absolute numbers of TCR γδ will however per se be higher in PBSC due to the overall higher T cell concentration compared to BM. To our knowledge our study is the first to compare TCR γδ subsets in BM and PBSC. Although not statistically significant probably due to the low number of observations, we found a trend towards lower fraction of Vδ2 cells and higher fraction of nonVδ1nonVδ2 in PBSC compared to BM. This could be due to the observed preferential mobilization of G-CSF on the nonVδ1nonVδ2 subtype. Similar associations were seen for HLA-DR positive TCR γδ cells which were more frequent in PBSC compared to BM and increased during G-CSF stimulation. Regarding NK cells, the percentage of these out of lymphocytes did not differ between PBSC and BM in this study. This finding was in opposition to a 2001 study which compared lymphocyte distribution in grafts from PBSC, BM and cord blood and found significantly increased fractions of NK cells in BM compared to PBSC (38). Within the NK cell compartment, the fraction of the mature effector CD56dim cell subset preserved by G-CSF was significantly higher in PBSC compared to BM in this study.

The concentrations of TCR γδ and NK cells in donors before G-CSF stimulation correlated with the corresponding cell doses in harvested grafts. This opens for the possibility of donor selection, should high graft doses of these innate effector cells be desired for HSCT with multiple donor choices, as well as for donor selection in the growing field of grafts designed for exploiting the specific potential of innate effector cells (39, 40).

In this study, the dosage of G-CSF is set at 10 µg/kg/day for 5 days for all donors. Different administration schedules could advantageously be studied in order to perhaps optimize mobilization of TCR γδ and NK cells. Moreover, the effects on TCR γδ and NK cell of another mobilizing agent, Plerixafor, are unknown and as this information could also help guide decisions on different mobilization strategies (41, 42).

The possibility to mobilize donors with G-CSF in order to obtain high numbers of TCR γδ and NK cells with polyclonal and activated phenotypes could also be interesting for the development of advanced immunotherapeutic approaches including redirection with chimeric antigen receptors (43). Another aspect for exploiting the effect of G-CSF in already established forms of cellular therapy is for donor lymphocyte infusions (DLI) (44, 45). DLI is used both prophylactic after HSCT and therapeutic in the case of post-transplant relapse and is usually harvested without prior G-CSF stimulation of the donor. Studies of the effect of G-CSF on TCR γδ and NK cells in DLI harvest could clarify if the potential GVL-effect of DLI could be enhanced by administration of G-CSF, perhaps in lower doses than for the PBSC harvest.

To our knowledge this is the largest study to date investigating the G-CSF effect on TCR γδ and NK cell subtypes in healthy donors for PBSC-HSCT and the first to include phenotype markers of differentiation and activation. Matched pre- and post G-CSF donor and corresponding graft samples provided additional information indicating preferential harvest efficiency between cell subtypes. Results from the latter are however limited by the number of donor/recipient pairs available for graft characterization and comparisons of graft types and need confirmation in larger studies.

In conclusion, G-CSF stimulation in healthy donors for PBSC-HSCT mobilized TCR γδ equally to TCR αβ cells, preferentially mobilized cells of the nonVδ2 types and increased the fraction of HLA-DR positive TCR γδ cells. G-CSF preserved NK cells of the CD56dim subset and increased the expression of the activating receptor NKG2D both of which have been associated with anti-tumor effects. These alterations could enhance the contribution of donor TCR γδ and NK cells to the GVL effect after transplantation and are important in an era of increasingly use of stimulated PBSC for manipulated grafts for HSCT (46). Also, knowledge of potentially G-CSF-induced modification of innate effector cells could drive decisions on the use of G-CSF stimulation for other advanced immunotherapeutic approached including adoptive cell therapy outside the HSCT setting.

## Data Availability Statement

The raw data supporting the conclusions of this article will be made available by the authors, without undue reservation.

## Ethics Statement

The studies involving human participants were reviewed and approved by Danish National Committee on Health Research Ethics (H-15005137). The patients/participants provided their written informed consent to participate in this study.

## Author Contributions

LM performed the research, analyzed the data, and wrote the manuscript. HM and LR contributed with new reagents and analytic tools in the laboratory and helped write the manuscript. HS, AF-N, and EH designed the research and helped write the manuscript. All authors contributed to the article and approved the submitted version.

## Funding

This study was supported by unrestricted grants from the Novo Nordisk Foundation (NNF15OC0014158) and the Danish National Research Foundation (grant 126).

## Conflict of Interest

The authors declare that the research was conducted in the absence of any commercial or financial relationships that could be construed as a potential conflict of interest.
