# Supplementary material for: Granulocyte Colony-Stimulating Factor Effectively Mobilizes TCR γδ and NK Cells Providing an Allograft Potentially Enhanced for the Graft-Versus-Leukemia Effect for Allogeneic Stem Cell Transplantation
Source: Front Immunol. 2021 Mar 10;12:625165. doi: 10.3389/fimmu.2021.625165 (PMC7988077; doi:10.3389/fimmu.2021.625165)

Supplemental data

Table 1. Two-tube panel of monoclonal antibodies (mAB) used for leukocyte phenotyping. Antibodies were titrated and used in saturating concentrations.

Flurochrome	mAB	Clone	Manufacturer catalog no.
**Tube 1**			
FITC	TCRαβ	Clone WT31	BD-333140
PE	TCRγδ	Clone 11F2	BD-333141
PerCp Cy5.5	CD4	Clone SK3	BD-332772
PE-Cy7	CD45RA	Clone L48	BD-337186
Alexa Fluor 647	CD197	Clone 150503	BD-560816
-			
APC-H7	CD45RO	Clone UCHL1	BD-561137
V450	HLA-DR	Clone L243	BD-655874
V500	CD3	Clone SP34-2	BD-560770
BV605	CD8	Clone SK1	BD-564116
**Tube 2**			
FITC	TCRVδ2	Clone 123R3	Miltenyi-130-095-798
PE	TCRγδ	Clone 11F2	BD-333141
-			
PE-Vio770	TCRVδ1	Clone REA173	Miltenyi-130-100-540
APC	CD314	Clone 1D11	BD-558071
-			
APC-H7	CD16	Clone 3G8	BD-560195
V450	CD56	Clone B159	BD-560360
V500	CD3	Clone SP34-2	BD-560770
BV605	CD337	Clone p30-15	BD-563384

Figure 1. Extracts from flow panels and gating strategies in tube 1 (page 3-4) and tube 2 (page 5-6). Dead cells/debris and dublets were removed as shown in the dot plot forward and side scatter (FSC/SSC) and FSC Area/FSC High. Lymphocytes were identified based on their forward and side scatter properties. In tube 1, CD3pos events were selected on the CD3/SSC dot plot and subsequintly CD3 T cells were separated in a TCR αβ/TCR γδ plot and TCR αβ T cells were further separated in a CD4/CD8 plot (not shown). TCR αβ T cells, TCR γδ T cells and CD4- and CD8 T cells were separately investigated for differentation markers in a CD45RA/CD45RO plot for identification of CD45RAneg/CD45RApos memory cell phenotypes, and a CD45RA/CD197 plot for identification of central(CD45RAnegCD197pos)/effector(CD45RAneg/CD197neg) memory-, CD45RApos/CD197pos naive-, and CD45RApos/CD197neg TEMRA cell phenotypes. The expression of HLA-DR were investigated in histograms for TCR αβ T cells, TCR γδ T cells and CD4- and CD8 T cells separately. In tube 2, TCR γδ (CD3pos) T cells were identified in a CD3/TCR γδ plot, and TCR γδ T cells were furthermore separated in subtypes in a TCR Vδ2/TCR Vδ1 plot. NK cells were identified in a CD56/CD16 plot gated on CD3neg lymphocytes and 2 populations (CD56bright and CD56dim) were identified based on CD56 and CD16 expression. TCR γδ T cells, TCR Vδ1 T cells, TCR Vδ2 T cells, CD56bright NK cells and CD56dim NK cells were separately investigated for the expression of CD314 and CD337 in histograms and analyzed based on fractions of cells positive as well as the MFI (mean fluorescence intensity) expression.

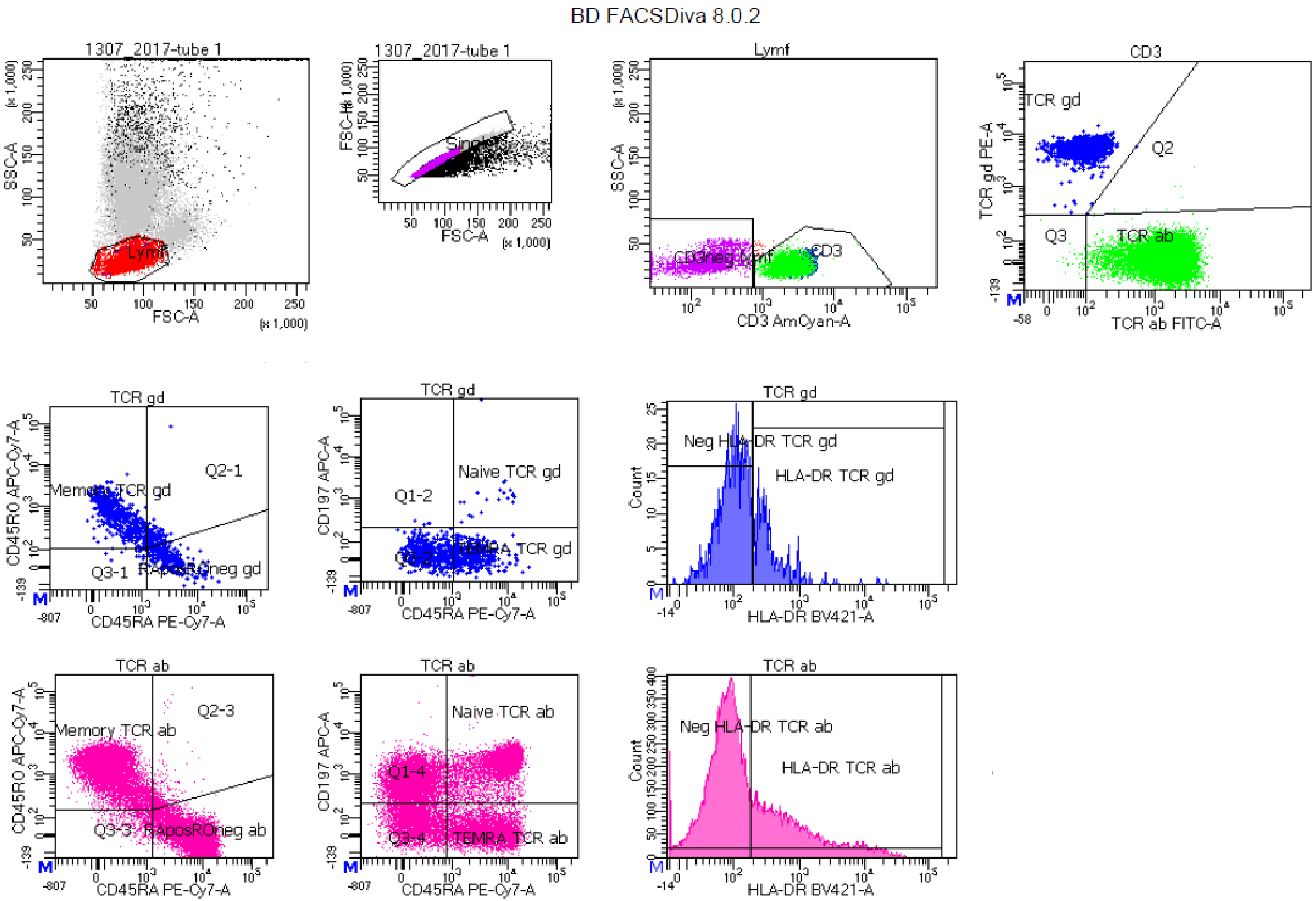
BD FACSDiva 8.0.2
1307_2017-tube 1
SSC-A
FSC-A
Lymf
Singlets
FSC-H
CD3
CD3neg Lymf
CD3 AmCyan-A
TCR gd
Q2
Q3
TCR ab
TCR gd PE-A
TCR ab FITC-A
Memory TCR gd
Q2-1
Q3-1
RAposROneg gd
CD45RO APC-Cy7-A
CD45RA PE-Cy7-A
Naive TCR gd
Q1-2
TEMRA TCR gd
CD197 APC-A
Neg HLA-DR TCR gd
HLA-DR TCR gd
Count
HLA-DR BV421-A
Memory TCR ab
Q2-3
Q3-3
RAposROneg ab
Naive TCR ab
Q1-4
Q3-4
TEMRA TCR ab
Neg HLA-DR TCR ab
HLA-DR TCR ab

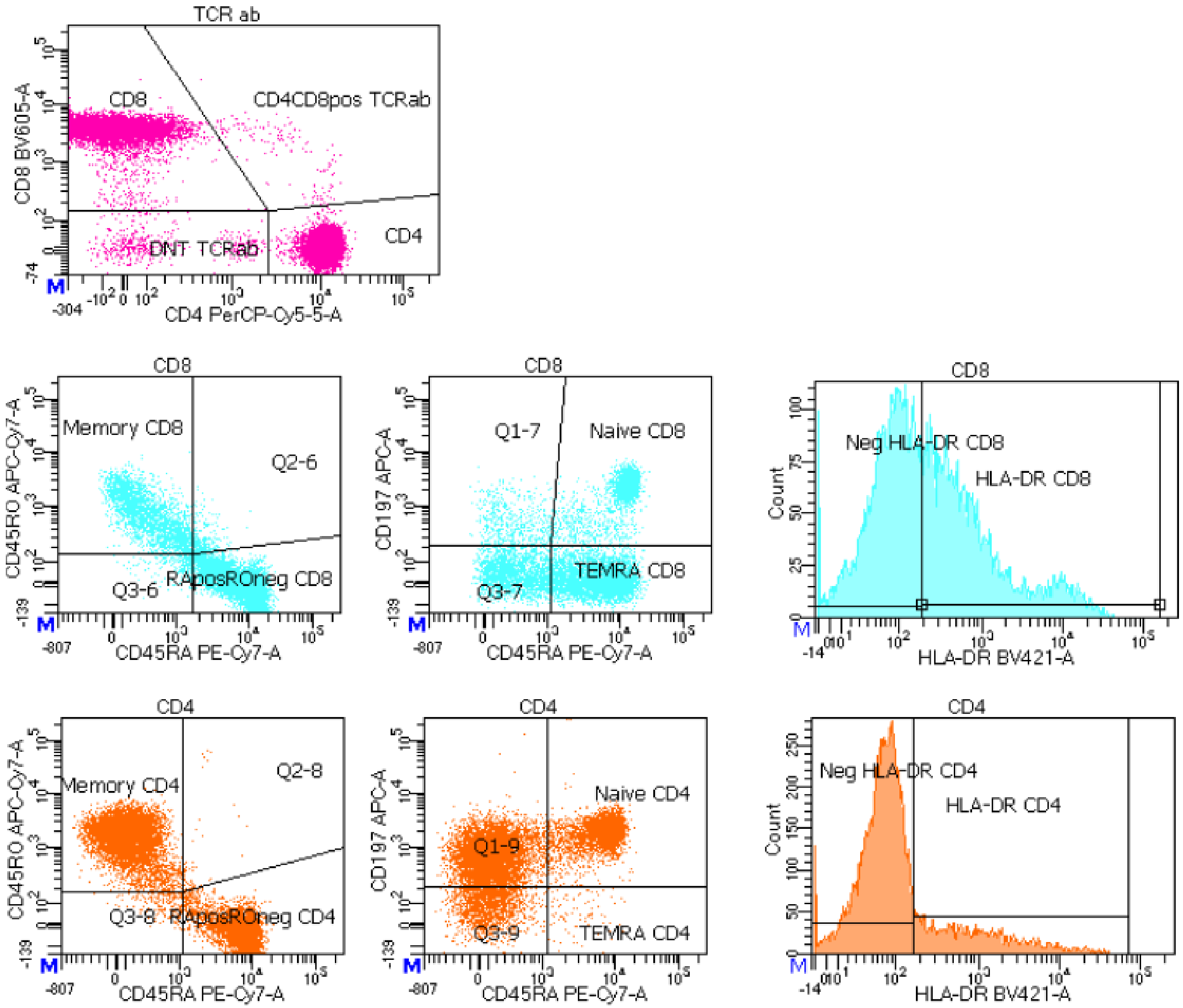

TCR ab
CD8
CD4CD8pos TCRab
DNT TCRab
CD4
CD8 BV605-A
CD4 PerCP-Cy5-5-A
CD8
Memory CD8
Q2-6
Q3-6
RAposROneg CD8
CD45RO APC-Cy7-A
CD45RA PE-Cy7-A
CD8
Q1-7
Naive CD8
Q3-7
TEMRA CD8
CD197 APC-A
CD45RA PE-Cy7-A
CD8
Neg HLA-DR CD8
HLA-DR CD8
Count
HLA-DR BV421-A
CD4
Memory CD4
Q2-8
Q3-8
RAposROneg CD4
CD45RO APC-Cy7-A
CD45RA PE-Cy7-A
CD4
Q1-9
Naive CD4
Q3-9
TEMRA CD4
CD197 APC-A
CD45RA PE-Cy7-A
CD4
Neg HLA-DR CD4
HLA-DR CD4
Count
HLA-DR BV421-A

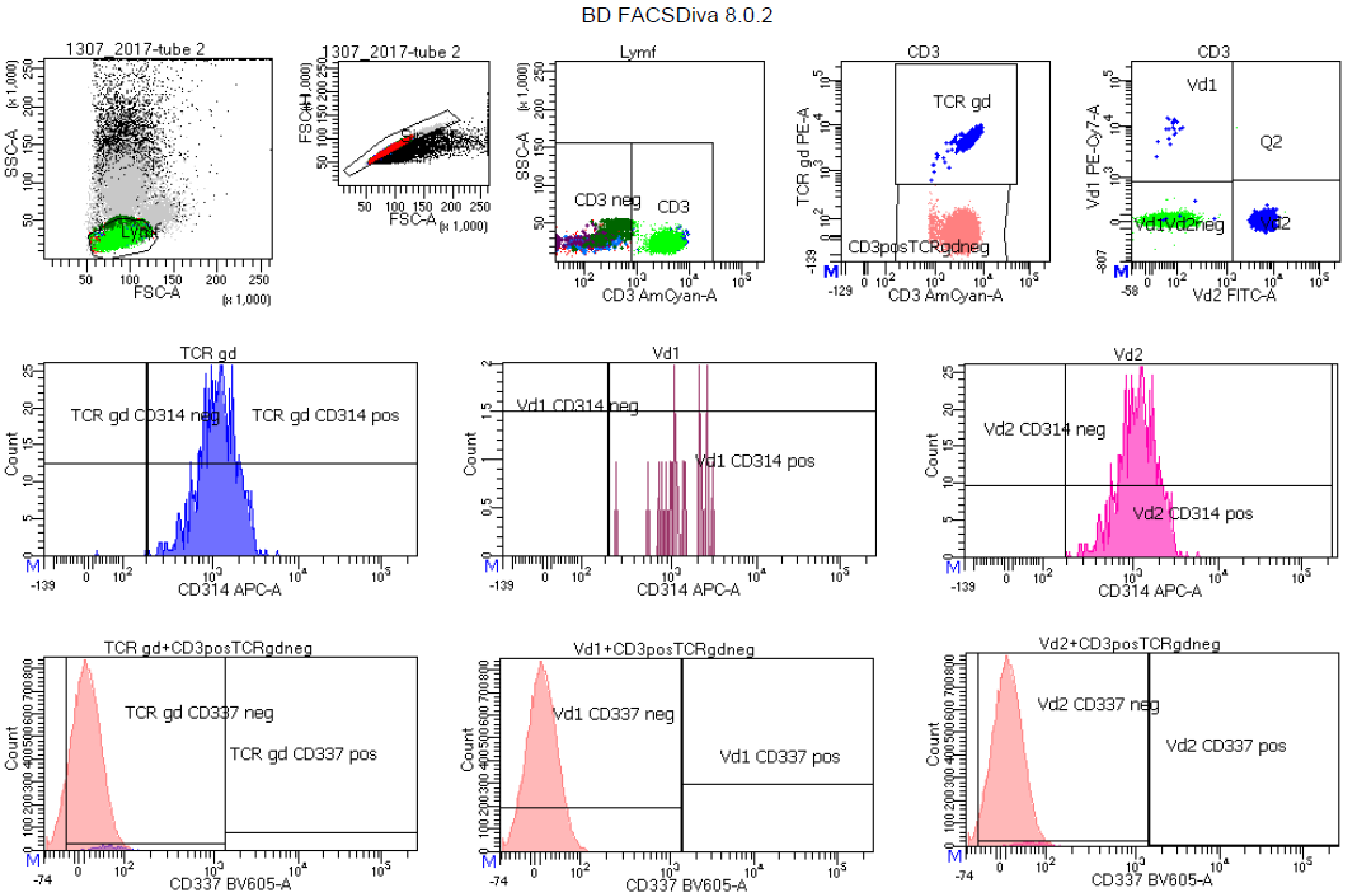
BD FACSDiva 8.0.2
1307_2017-tube 2
SSC-A
FSC-A
Lymf
FSC-H
CD3 neg
CD3
SSC-A
CD3 AmCyan-A
TCR gd
CD3posTCRgdneg
TCR gd PE-A
Vd1
Q2
Vd1Vd2neg
Vd2
Vd1 PE-Cy7-A
Vd2 FITC-A
TCR gd CD314 neg
TCR gd CD314 pos
Count
CD314 APC-A
Vd1 CD314 neg
Vd1 CD314 pos
Vd2 CD314 neg
Vd2 CD314 pos
TCR gd+CD3posTCRgdneg
TCR gd CD337 neg
TCR gd CD337 pos
CD337 BV605-A
Vd1+CD3posTCRgdneg
Vd1 CD337 neg
Vd1 CD337 pos
Vd2+CD3posTCRgdneg
Vd2 CD337 neg
Vd2 CD337 pos

BD FACSDiva 8.0.2

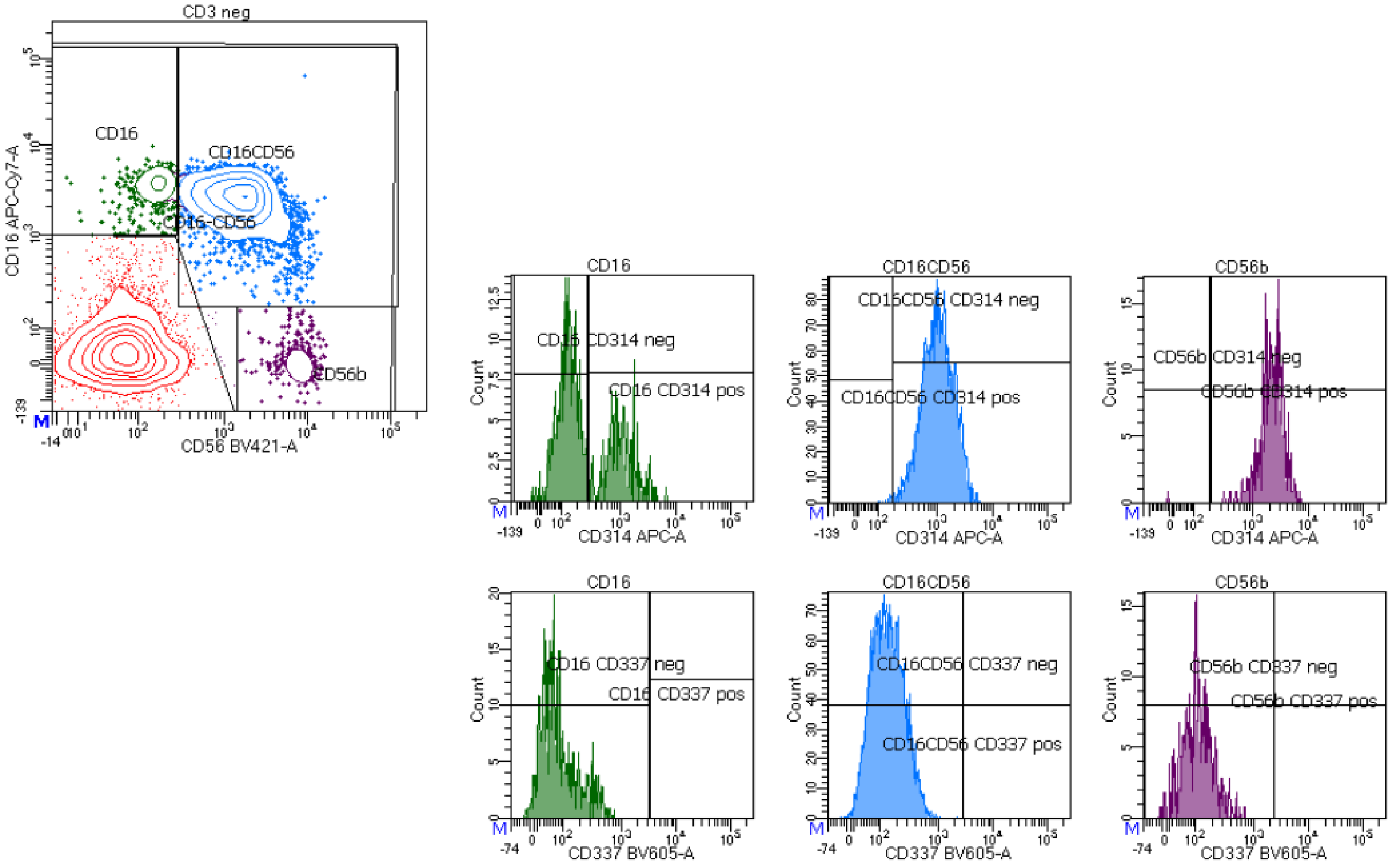

Table 2. Subset definitions and phenotypes. The absolute concentrations of CD3, CD4, CD8 and NK cells were calculated by the BD ™ Trucount system. Additional fractions and concentrations were calculated from the panels in Table 1.

Subset definition	Phenotype
CD3+TCR γδ+	TCR γδ cells
CD3+TCR γδ+CD45RA+CD197+	Naive TCR γδ cells
CD3+TCR γδ+CD45RA-CD197+	Central memory TCR γδ cells
CD3+TCR γδ+CD45RA-CD197-	Effector memory TCR γδ cells
CD3+TCR γδ+CD45RA+CD197-	TEMRA TCR γδ cells
CD3+TCR γδ+Vδ1+	TCR Vδ1 cells
CD3+TCR γδ Vδ2+	TCR Vδ2 cells
CD3+TCR γδ+Vδ1-Vδ2-	TCR nonVδ1-nonVδ2
CD3+TCR αβ+CD4+	CD4 T cells
CD3+TCR αβ+ CD4+CD45RA+CD197+	Naive CD4 T cells
CD3+TCR αβ+ CD4+CD45RA-CD197+	Central memory CD4 T cells
CD3+TCR αβ+ CD4+CD45RA-CD197-	Effector memory CD4 T cells
CD3+TCR αβ+ CD4+CD45RA+CD197-	TEMRA CD4 T cells
CD3+TCR αβ+CD8+	CD8 T cells
CD3+TCR αβ+ CD8+CD45RA+CD197+	Naive CD8 T cells
CD3+TCR αβ+ CD8+CD45RA-CD197+	Central memory CD8 T cells
CD3+TCR αβ+ CD8+CD45RA-CD197-	Effector memory CD8 T cells
CD3+TCR αβ+ CD8+CD45RA+CD197-	TEMRA CD8 T cells
CD16/CD56+	NK cells
CD56++CD16low/neg	CD56bright NK cells
CD56+CD16+	CDCD56dim NK cells

Figure 2. Extracts from flow panels showing a) before- and b) after-G-CSF plots of TCR γδ and NK cell subtypes in donor #46. The Vδ1 percent increased from 33% to 49%, and the Vδ2 percent decreases from 50% to 44%. In this donor, there was a decrease in the nonVδ1nonVδ2 subset from 17 to 7% (calculated as total TCR γδ cells minus [Vδ1+Vδ2 cells]). The percentages of NK cells subtypes remained stable in this donor which was comparable to the overall data.

a) TCR γδ cell subtypes before G-CSF

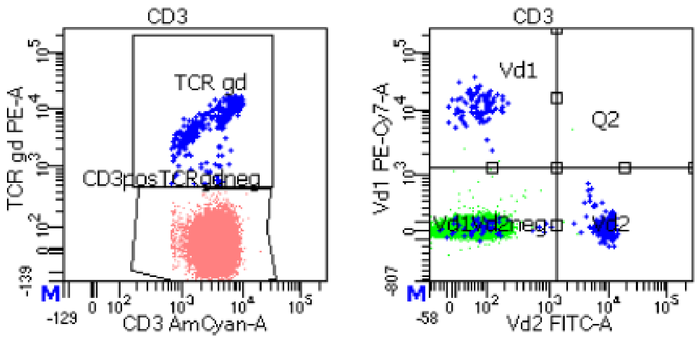

NK cell sybtypes before G-CSF

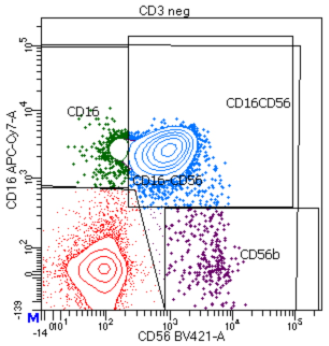

b) TCR γδ cell subtypes after G-CSF

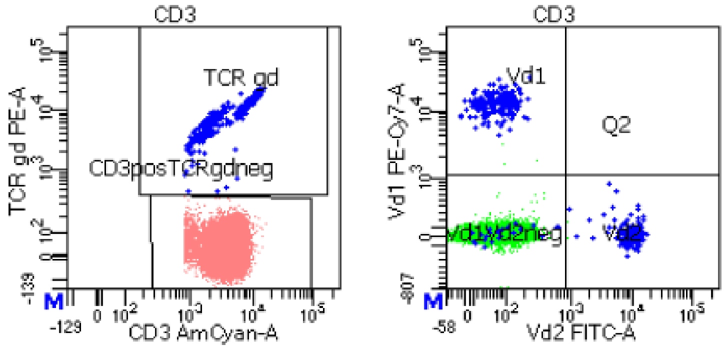

NK cell sybtypes after G-CSF

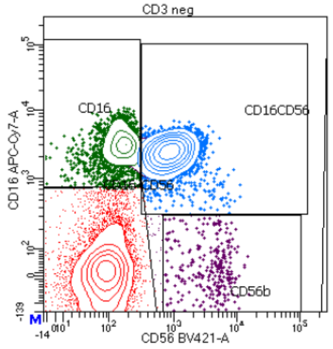

Figure 3. Scatter plot of TCR γδ cell concentrations in donor pre-G-CSF blood samples and TCR γδ cell concentrations in corresponding graft samples. Kendall's tau-b test for correlation: Rho 0.71, p<0.001.

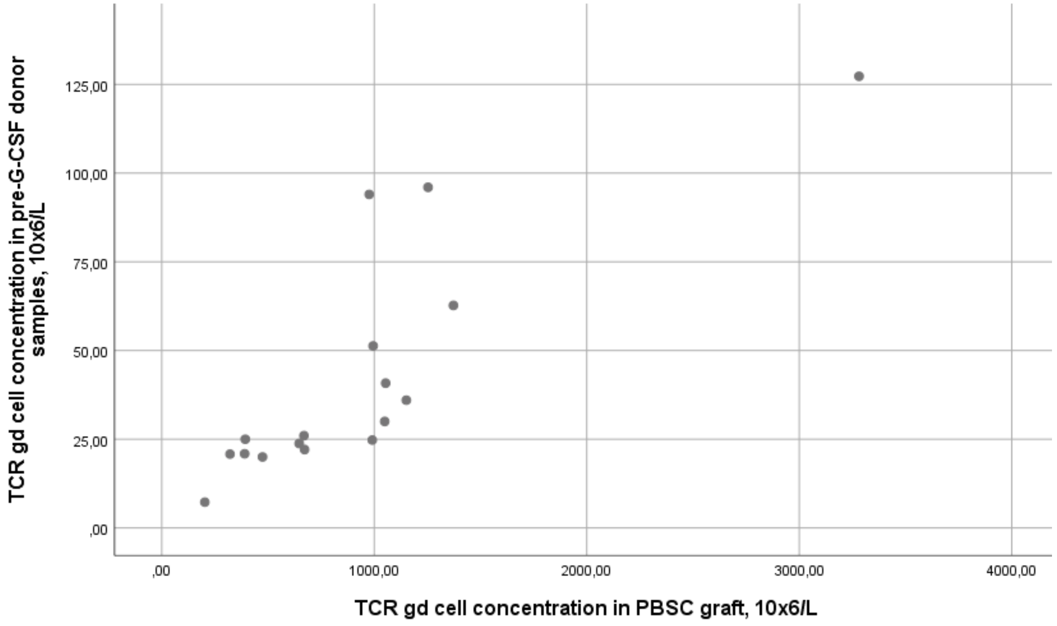

Supplement: Supplementary file 1 [file DataSheet_1.pdf]
